# The Impact of Sepiolite on Sensor Parameters during the Detection of Low Concentrations of Alcohols

**DOI:** 10.3390/s16111881

**Published:** 2016-11-09

**Authors:** Patrycja Suchorska-Woźniak, Olga Rac, Marta Fiedot, Helena Teterycz

**Affiliations:** Faculty of Microsystem Electronics and Photonics, Wrocław University of Science and Technology, Janiszewskiego 11/17, Wrocław 50-372, Poland; olga.rac@pwr.edu.pl (O.R.); marta.fiedot@pwr.edu.pl (M.F.); helena.teterycz@pwr.edu.pl (H.T.)

**Keywords:** nanomaterial, sepiolite filter, alcohols, gas detection, resistive gas sensors

## Abstract

The article presents the results of the detection of low-concentration C1–C4 alcohols using a planar sensor, in which a sepiolite filter was applied next to the gas-sensitive layer based on tin dioxide. The sepiolite layer is composed of tubes that have a length of several microns, and the diameter of the single tube ranges from several to tens of nanometers. The sepiolite layer itself demonstrated no chemical activity in the presence of volatile organic compounds (VOC), and the passive filter made of this material did not modify the chemical composition of the gaseous atmosphere diffusing to the gas-sensitive layer. The test results revealed that the structural remodelling of the sepiolite that occurs under the influence of temperature, as well as the effect of the filter (a compound with ionic bonds) with molecules of water, has a significant impact on the improvement of the sensitivity of the sensor in relation to volatile organic compounds when compared to the sensor without a filter.

## 1. Introduction

As is well known, chemical resistive gas sensors consist of two basic elements—a receptor part and an actuator part. The receptor part (gas-sensitive) consists of polycrystalline metal oxides that have semiconductor properties. These sensors operate at an elevated temperature and during catalytic detection the gas-sensitive materials are exposed to an atmosphere with an extremely variable composition. For this reason, the electrical characteristics of the resistive gas sensors not only change as a result of the interaction with determined gas molecules, but also as a result of the reaction with the molecules of other gases present in the gaseous atmosphere. If the physico-chemical properties of these gases are similar to the properties of the determined gas, they intensively affect the electrical parameters of the gas-sensitive material. For this reason, regardless of the material of the receptor layer, selectivity and stability still remain at a level that is insufficient for many applications [1,2,3]. Moreover, the semiconductor material of the receptor layer gradually loses sensitivity due to the irreversible adsorption of trace impurities of the atmosphere. This process is called “poisoning” and locks the surface active sites, which in turn causes a drift of the electrical parameters as a function of time (poor long-term stability) [3,4].

One of the most promising techniques to improve the performance parameter characteristics of the sensors, especially selectivity, is to build sensor structures that consist of at least two layers that have different electrical and catalytic properties. A layer that has direct contact with the electrodes acts as a proper active layer, while other materials applied to it act as a filter that modifies the sensor parameters. These additional layers are most often used to eliminate or reduce the impact of water on sensor readings. By constructing multi-layer sensors, it is possible to obtain a significant improvement in their performance parameters [5]. In addition, limitations in the fabrication of the resistive gas sensors that have satisfactory performance characteristics also result from the complex physical and chemical processes that are difficult to analyse and occur in these types of sensors.

The literature presents various constructions of multi-layer resistive sensors with both physical (passive) filters and chemical (active) filters. Active filters most often consist of thin layers of metal that have a high catalytic activity, while the layers of activated carbon or zeolites are frequently used as physical filters [4,6,7]. Due to the specific properties of sepiolite [8], such as a large, specific surface area, a large adsorption capacity and a small channel diameter, this compound can be successfully used as a physical filter to improve sensor sensitivity and selectivity [8,9,10].

The structure of the gas-sensitive material and the applied filter plays an important role in identifying the mechanism of the interaction of the materials with the detected gases. Knowledge of this mechanism, as well as the assessment of the impact of the additional presence of water molecules on the sensitivity of the sensors is important, from a medical point of view, among others. The article presents the detection of alcohols, some of which are present at low concentrations in the air exhaled by a human [11,12]. Significantly exceeded thresholds of these compounds may indicate metabolic disorders or lead to the emergence of cross sensitivity with respect to, among others, volatile sulphur compounds, whose determination in exhaled air is important for people who suffer from halitosis [13]. Reinforcing or muting the sensors’ response at the expense of one gas and in favour of another gas can improve selectivity and eliminate the interfering gases.

This article presents the studies on the planar sensor made by the thick-film technology, in which a layer of sepiolite—a natural aluminosilicate—was printed on a layer of gas-sensitive material made of tin dioxide. This physical filter significantly influenced the parameters of the sensor. The response of the developed sensors in atmospheres containing selected C1–C4 alcohols at a concentration of 1 to 5 ppm was examined, and the first attempt to analyse the phenomena affecting the operational parameters of the sensor with the sepiolite filter was made.

## 2. Materials and Methods

Sensors were made on a substrate of alundum ceramics, which have a thickness of 250 μm. On the one side of the substrate, a meander shaped platinum heater and gold contacts (ESL 8846-G, ESL Europe, Reading, UK) forming the electrical supply to the heater were printed. The dimensions of a single support structure of the sensor are 25.40 × 2.45 mm^2^, while the meander shaped platinum area is approx. 7.10 mm^2^.

**Figure 1:**
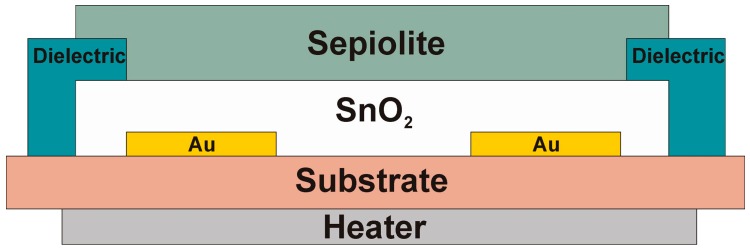
The cross-section of the structure of the thick-layered gas sensor based on tin dioxide (IV) with a sepiolite filter.

On the other side of the substrate, gold electrodes were made, onto which a layer of tin dioxide with a thickness of about 40 μm was printed. Then, the dielectric (ESL 4913-G, ESL Europe, Reading, UK) was printed onto the electrode and partly onto the gas-sensitive layer. The dimensions of the gas-sensitive layer not covered by the dielectric amounted to 800 × 1200 μm^2^. The distance between the electrodes was approximately 250 μm. A sepiolite layer with a thickness of approximately 40 μm and dimensions of 800 × 1600 μm^2^ was printed onto the exposed layer of tin dioxide and partly onto the dielectric. The sensor layers, presented in Figure 1, were printed in such a manner that the gas-sensitive material, i.e., tin oxide, had no direct contact with the gas atmosphere.

Pastes designed for the construction of the sensor were prepared independently. The synthesis of the modified SnO_2_ was performed by Okazaki’s method [14]. The obtained SnO_2_ powder was fired at 600 °C for 30 min and then mixed with the organic vehicle (ESL-400, ESL Europe, Reading, UK) and triturated in an agate mortar until a paste with suitable rheological properties was obtained. The paste of the specific material was printed twice, by drying the layers after each printing, first at room temperature and then at 125 °C for 10 min. A contact physical filter made from sepiolite was directly printing onto the gas-sensitive layer using also the standard thick-film technology. The complete structure of the sensor was fired at 850 °C for 2 h.

Analysis of the powders was performed using SEM, a scanning electron microscope LEO 435 VP (Carl Zeiss Inc, Oberkocken, Germany), equipped with the EDS, X-ray microanalysis system (Roentec, Berlin, Germany). The studies of the sepiolite microstructure were conducted using TEM, a transmission electron microscope Tecnai G2 20 X-TWIN with EDAX X-ray microanalyzer (FEI Company, Hillsboro, OR, USA).

**Figure 2:**
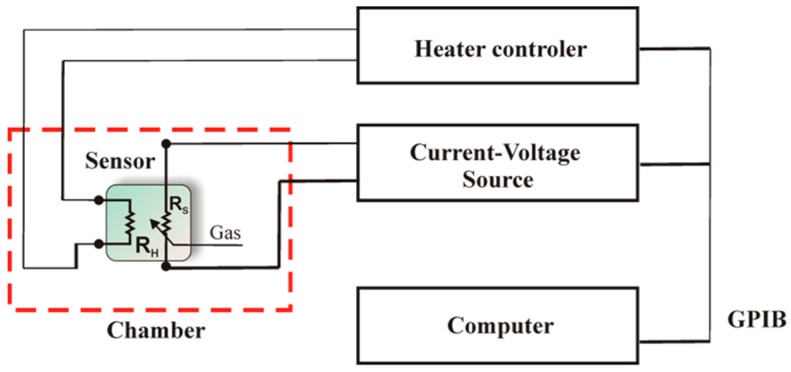
Schematic diagram of the construction of the measuring station used during the characterization of the sensors by the temperature-stimulated conductivity and voltammetric techniques.

Since the kinetics of the physico-chemical processes occurring on the surface of the gas-sensitive materials is strongly dependent on the temperature, sensor characterization was performed by a method known as Temperature Stimuled Conductance (TSC) and by electrochemical methods as a function of temperature. The TSC method involves measuring the conductance of the test sensor temperature. When measuring using the TSC method, a tested sensor was placed in the measuring chamber, as illustrated in Figure 2, containing a gaseous atmosphere (pure synthetic air with humidity of 30% RH with/without alcohol) of the well-defined composition. The operating temperature of the heater was periodically changed linearly at a constant speed of 2°/s in a range from 150 °C to 750 °C. The current flowing through the gas-sensitive material was recorded during both the temperature rise and drop. The gas-sensitive structure was polarized in DC voltage using the Keithley 2400 current-voltage source (Keithley Instruments Inc., Cleveland, OH, USA) and the electric current was measured. Then, using the Ohm’s law, the resistance and conductance of the gas-sensitive material was determined. Electric measurements were carried out at a test stand comprising a HP E3632 A power supply unit type (Agilent Technologies Inc., Santa Clara, CA, USA), a Keithley 2400 current–voltage source, the measurement chamber and a PC provided with appropriate software. Current-voltage characteristics of sensors at various temperatures were recorded using an potentiostat-galvanostat type SI 1287 from Solartron Analytical (Farnborough, UK), with the use of the CorrWare software from Scribner Associates Inc. (Southern Pines, NC, USA) [15].

On the basis of the temporary temperature changes and current, the baseline characteristics were determined for the temperature changes in the conductivity of the tested sensors. On this basis, the temperature changes of sensitivity were then determined, which were defined as the relationship of the conductance of the sensor in an atmosphere containing the determined gas and pure synthetic air with humidity of 30% RH to the conductance in pure synthetic air with humidity of 30% RH Equation (1).

(1) $$S_{gas}=\frac{G_{gas}(T)}{G_{0}(T)}$$

To determine the impact of the sepiolite filter layer on the parameters of the sensor with the active layer composed of tin dioxide, electrical characterization of the following sensors was performed:

- with a layer made only from sepiolite (without gas sensitive layer of tin dioxide),
- with a layer made of undoped tin dioxide,
- double layered with a gas-sensitive layer made of undoped tin dioxide (active layer) and a natural filter made of sepiolite.

## 3. Results and Discussion

### 3.1. Properties of the Sepiolite Filter

**Figure 3:**
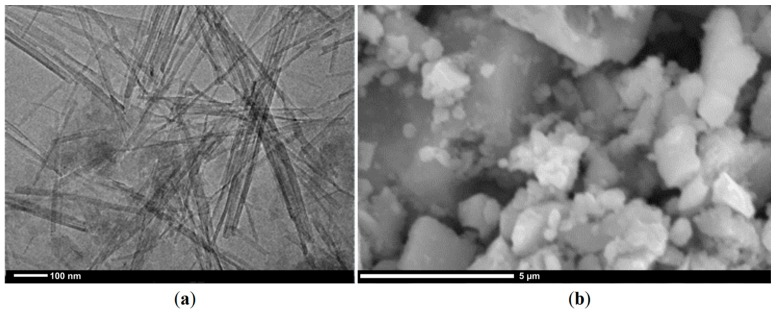
The microstructure of: (**a**) the sepiolite; (**b**) the SnO_2_ layer.

The research refers to a sensor with a passive (physical) filter, which was the sepiolite layer—a natural aluminosilicate. The analysis of the chemical composition of the sepiolite showed that the basic oxides which form part of the sepiolite include silicon oxide (IV) in an amount of 65.64 wt %; magnesium oxide in an amount of 34.02 wt % and small amounts of aluminum oxide and calcium oxide, respectively, in amounts of 0.10 wt % and 0.25 wt %. As the studies performed with the TEM method demonstrated, the sepiolite layer is composed of tubes that have a length of several microns what was shown in Figure 3a. The diameter of the single tube ranges from several to tens of nanometers. In contrast, presented in Figure 3b, the gas-sensitive layer of tin dioxide consisted of grains formed by a cluster of crystallites.

**Figure 4:**
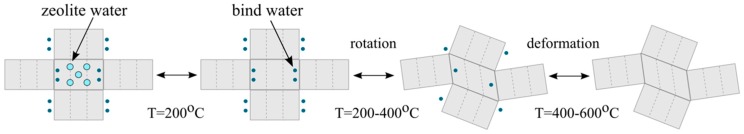
Changes in the structure of the sepiolite under the influence of temperature [16].

Literature data indicates that along with a temperature increase, a gradual change in the crystalline structure of the sepiolite occurs, as presented in Figure 4 [16]. The channels in the structure of the sepiolite close, causing a decrease in the specific surface of the sepiolite, which can affect the diffusion rate of the substrates to the surface and the reaction products from the surface of the gas-sensitive layer.

**Figure 5:**
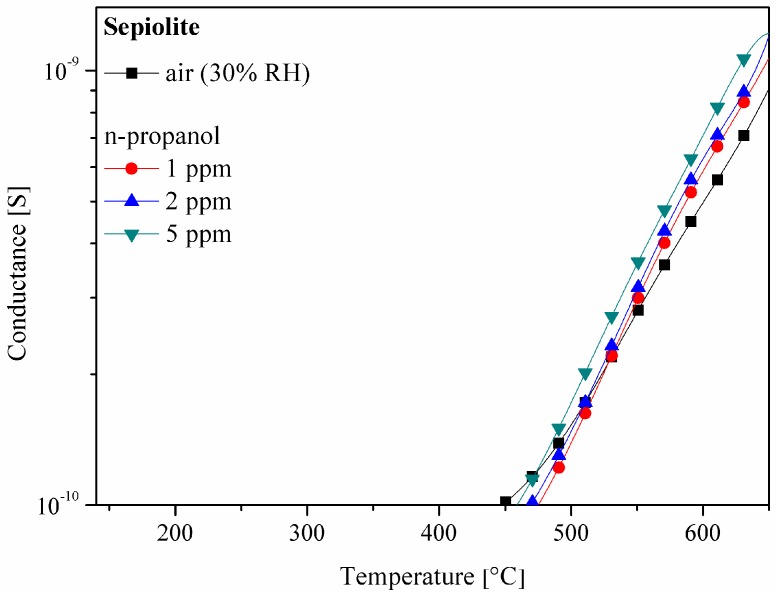
The effect of the composition of the atmosphere on the temperature changes of the conductance in the sepiolite layer. According to the catalogue data, the lower range of the Solartron SI 1287 m used was 0.4 nA.

Measurements of the sensor with a layer of sepiolite only (without active layer), using the TSC method in air and in an atmosphere with various concentrations of n-propanol, as presented in Figure 5, showed that sepiolite is a good isolator. For this reason, the tin oxide/sepiolite contact does not affect the parameters of the gas-sensitive layer. Due to the high resistivity of the sepiolite, its current-voltage characteristics in the temperature range from 300 °C to 500 °C were not determined. Similar difficulties with the determination of the current-voltage characteristics of sepiolite were also signalled in [17], the authors of which believe that the large resistivity of the sepiolite is the result of the random arrangement of fibers. The introduction of Fe^2+^ ions into the sepiolite and the orientation of the fibers in the magnetic field reduce its resistivity. When the sensor temperature was higher than 450 °C, illustrated in Figure 5, it was possible to determine the resistance of the sepiolite layer.

The presence of n-propanol did not produce significant changes in conductance over the range of temperatures. Minor changes in conductance are due to changes of atmosphere composition caused by thermal degradation of alcohols at high temperatures. The composition of the atmosphere slightly affects the high temperature parameters of dielectrics. On this basis, it was considered that the sepiolite layer does not show chemical activity in the presence of the alcohol and the passive filter made from this material will not modify the chemical composition of the atmosphere of the gas diffusing to the gas-sensitive layer. Moreover, the observed increase in the conductivity of the probe temperature above 450 °C is due to the increase in the electrical conductivity of the sepiolite, caused by the desorption of water and a change in the microstructure of the material [7,18]. Fripiat et al. [19] found that the specific surface of the sepiolite increases to approximately 300 m^2^/g at 100 °C, and then decreases when increasing the temperature, reaching 150 m^2^/g at 500 °C. Similar results were obtained in [7], where changes were discovered in the specific surface of the sepiolite of 263 m^2^/g to 60 m^2^/g in the temperature range of 100 °C to 900 °C. The increase in the surface area of the sepiolite layer in the initial temperature range would certainly affect the growth rate of the chemical process if it occurred on its surface. However, electrical tests, illustrated in Figure 5, showed that the filter material is not chemically active, since its conductance does not depend on the composition of the ambient atmosphere.

**Figure 6:**
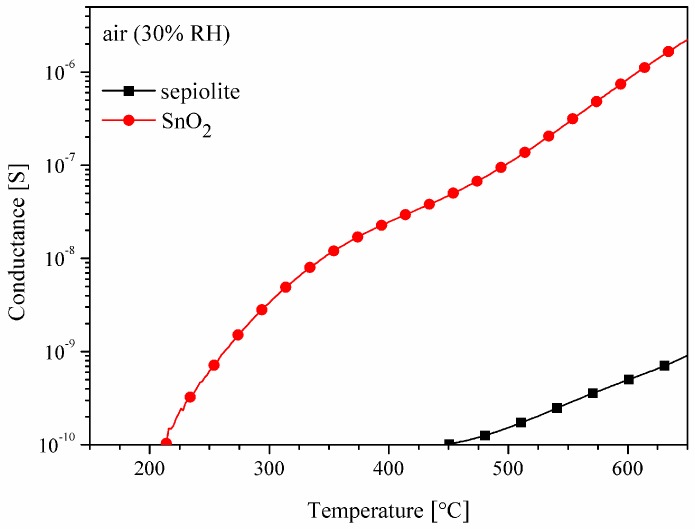
Thermal changes in the conductance of the sensors with the SnO_2_ layer or only the sepiolite layer in synthetic air.

By comparing the conductance of the sepiolite layer with the conductance of the tin dioxide layer with the same geometrical dimensions, it is clear that in the presented range of the operating temperature (Figure 6) of the resistive gas sensor the conductance of the SnO_2_ layer is much greater than the conductance of the sepiolite layer.

Accordingly, in the two-layer SnO_2_/sepiolite sensor, the current will only flow through the gas-sensitive layer of SnO_2_.

### 3.2. Current-Voltage Measurements of SnO_2_ and SnO_2_/Sepiolite Sensor

The tested resistive sensors with filters are multilayer structures and built from materials with different electrical and catalytic properties. Therefore, on the boundary of each layer, and between the grains within a given layer there can occur various electrical phenomena (rectifying or ohmic contacts), various chemical reactions, and various physical processes (diffusion), which affect the value of the sensor conductance.

**Figure 7:**
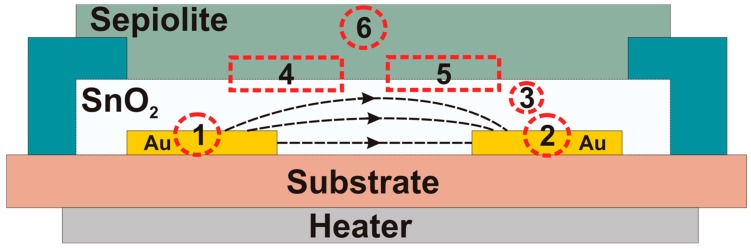
The cross-section of the structure of a thick-layered gas sensor based on tin dioxide (IV) with a sepiolite filter with indicated phase borders.

Considering the construction of the sensor with an active layer and a sepiolite filter, we can distinguish the following relations in its construction presented in Figure 7:

1 Metal/metal oxide formed at the phase boundary between the gold electrode and a gas-sensitive material (Au/SnO_2_—area No. 1), (SnO_2_/Au—area No. 2),
2 Metal oxide/metal oxide occurring inside the gas-sensitive layer between the individual grains (SnO_2_/SnO_2_—area No. 3),
3 Metal oxide/sepiolite, created as a result of the imposition of a passive filter layer (SnO_2_/sepiolite—area No. 4), (sepiolite/SnO_2_—area No. 5),
4 Sepiolite/sepiolite, occurring between the fibers of the filter (area No. 6).

**Figure 8:**
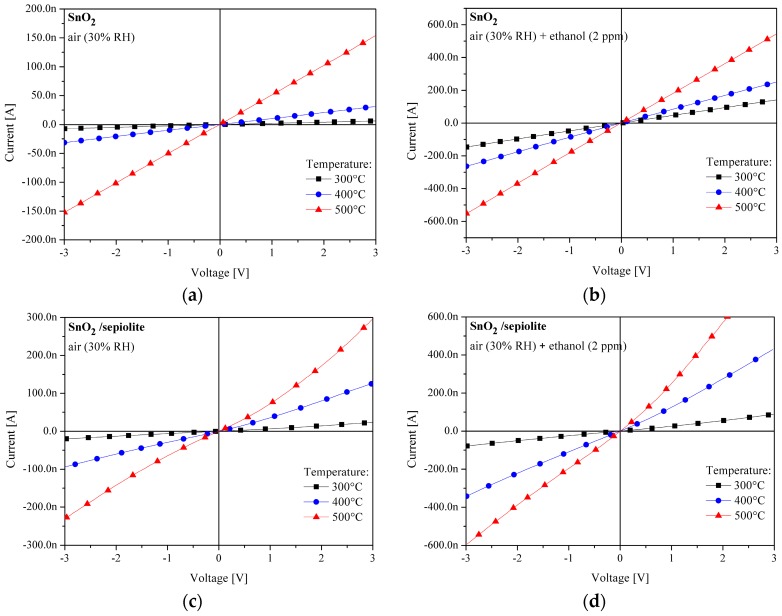
Current-voltage characteristics of the resistive gas sensor; (**a**) SnO_2_ in air (30% RH); (**b**) SnO_2_ in 2 ppm ethanol+air (30% RH); (**c**) SnO_2_/sepiolite in air (30% RH); (**d**) SnO_2_/sepiolite in 2 ppm ethanol + air (30% RH). ΔU = 150 mV/s.

The voltage and the direction of the structure’s polarity, may have an effect on the sensor’s output signal in sensors with heterocontact of different materials. To determine the effect of the individual heterocontacts on the parameters of the sensors, current-voltage measurements of the tested sensors (with and without sepiolite) were performed. The measurements were carried out in a range of 300 °C to 500 °C in air with a relative humidity of 30%, and in an atmosphere containing beside air (30% RH) also 2 ppm ethanol. Results of the sensor layer made of SnO_2_ and SnO_2_ with sepiolite filter, presented in Figure 8, demonstrated that the dependency of current on voltage (in a range of −3 V to 3 V) is linear, irrespective of the sensor temperature and the composition of the analysed atmosphere. The character of changes for all alcohols was the same.

These parameters only affect the level of current flowing through the structure and it is therefore considered that gold/tin oxide contacts are ohmic and do not affect the sensor parameters.

### 3.3. C1–C4 Alcohol Detection Using SnO_2_ and SnO_2_/Sepiolite Sensor

**Figure 9:**
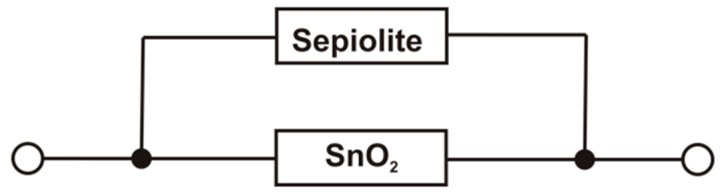
An equivalent electric circuit of the structure of the resistive gas sensor with a passive sepiolite filter.

By analysing the structure of the resistive gas sensor with the sepiolite filter and the results of the electrical characterization of the sensors with the single layer, it may be assumed that the sensor consists of two parallel resistors, representing individual layers (Figure 9).

The equivalent conductance ($G_{Sensor}$) of that system can be described by Equation (2):

(2) $$G_{Sensor}=G_{SnO_{2}}+G_{Sep}$$

in which:

$G_{SnO_{2}}$ is the conductance of the active layer,
$G_{Sep}$ is the conductance of the sepiolite layer.

**Figure 10:**
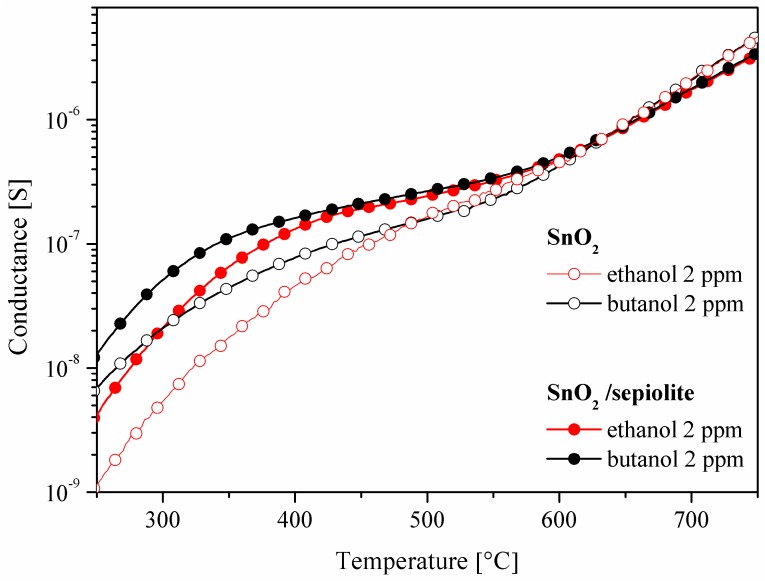
Temperature changes in the conductance of the SnO_2_ sensor with and without the sepiolite filter in 2 ppm of ethanol or butanol.

Since the conductance of the sepiolite layer is many times smaller than the conductance of the gas-sensitive layer ($G_{SnO_{2}}>>G_{sep}$), the conductance of the dual-layer sensor should be comparable to the conductance of the sensor built only from the layer of tin dioxide ($G_{Sensor}≅G_{SnO_{2}}$). However, measurements conducted in the atmosphere containing vapours of alcohols showed that dual-layer sensor has a higher conductance, due to the impact of water on the measurement (Figure 10), which is discussed in the next subsection.

**Figure 11:**
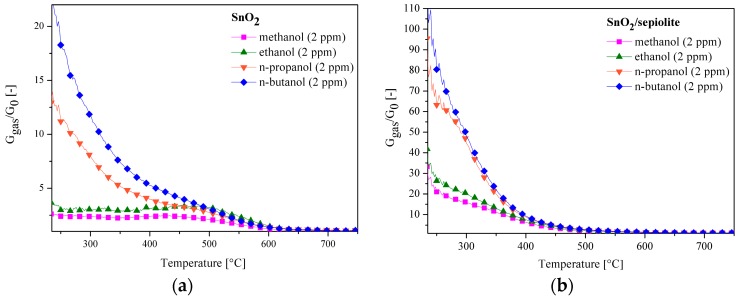
Temperature changes of the SnO_2_ sensor sensitivity: (**a**) without a filter; (**b**) with a filter in the atmosphere with a different composition (air with 30% RH + alcohol).

On the basis of temperature changes in the conductance of the sensors in the gas atmosphere with various compositions, the dependence of the sensitivity of these sensors over the entire temperature range was determined. It was found that the sensitivity of the sensor without a filter is lower than the sensitivity of the SnO_2_ sensor with the sepiolite filter as presented in Figure 11. The sensitivity of the sensor with a filter in the presence of n-butanol and n-propanol significantly increased. The optimum working temperature of the sensor also changed. The sensitivity of the sensor without a filter as a function of the type of gas at a temperature of 450 °C changes as follows: S_n-butanol_ > S_n-propanol_ > S_ethanol_ > S_methanol_ (Figure 11a). The sensitivity of the sensor with a filter changes in a similar manner, but it is much higher at 200 °C (Figure 11b). The sensitivity of the sensor with a filter above 500 °C was not dependent on either the temperature or the composition of the gas atmosphere.

**Figure 12:**
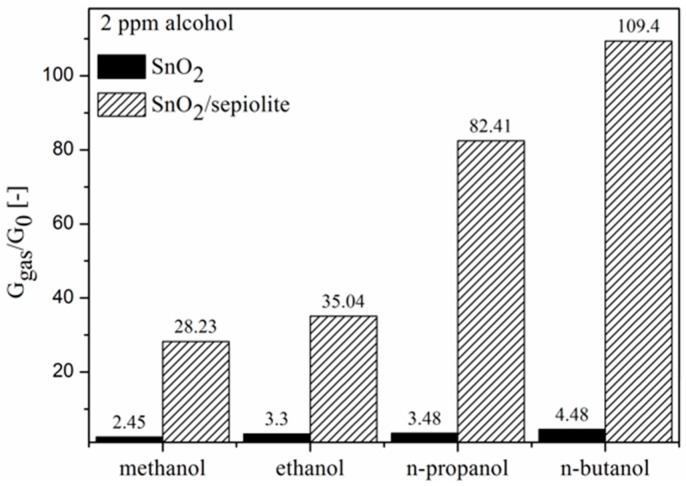
A comparison of the sensitivity of the two sensors on 2 ppm alcohol (air 30% RH).

As shown in Figure 12, by comparing the sensitivity of both sensors on volatile organic compounds, it can be noticed that the application of the sepiolite filter significantly improved the sensitivity of the sensor and enabled the detection of alcohol at lower temperatures. Furthermore, sensitivity significantly increased relative to the alcohols containing 3 and 4 carbon atoms.

The generally accepted mechanism of resistive gas sensor work is based on the series of physicochemical processes which occurs on the surface of semiconductor metal oxide materials [20,21]. In the case of such gas sensing materials as tin dioxide, the first step is chemisorption of oxygen molecules on surface vacancies. During the chemisorption, oxygen is taking electrons from the semiconductor sensor layer, causing the decrease in conductance of the layer, since SnO_2_ is an n-type semiconductor. As a result of chemisorption, the concentration of surface oxygen ions increases. Their degree of oxidation, and thus reactivity, depends on the temperature (3)–(6).

(3) O2(gas)↔ T1 O2(ads)

(4) O2(ads)+e−↔ T2 O2−

(5) O2−+e−↔ T3 2O−

(6) O−+e−↔ T4 O2−

where $T_{1}<T_{2}<T_{3}<T_{4}$.

In the temperature range from 200 °C to 520 °C, i.e., the range in which the highest sensitivity of the sensors is observed, the dominant ion form of the chemisorbed oxygen on the surface of SnO_2_ is the ion $O^{-}$ [20,22]. Thus, in the atmosphere of the determined volatile organic compounds, the following reactions can occur on the surface of the sensor material (7)–(10):

(7) $$\text{methanol }CH_{3}OH+3O^{-}\to CO_{2}+2H_{2}O+3e^{-}$$

(8) $$\text{ethanol }CH_{3}CH_{2}OH+6O^{-}\to 2CO_{2}+3H_{2}O+6e^{-}$$

(9) $$\text{n-propanol }CH_{3}CH_{2}CH_{2}OH+9O^{-}\to 3CO_{2}+4H_{2}O+9e^{-}$$

(10) $$\text{n-butanol }CH_{3}CH_{2}CH_{2}CH_{2}OH+12O^{-}\to 4CO_{2}+5H_{2}O+12e^{-}$$

During testing, the partial gas pressure of the oxygen in the determined gas atmosphere was constant. It is therefore concluded that the oxidation rate of the tested organic compounds at a fixed temperature should only depend on the determined compound and its partial gas pressure. Slightly higher conductance of the sensor with a filter in the atmosphere of the synthetic air can result from the impact of the filter on the rate of diffusion of:

- oxygen to the surface of tin dioxide,
- the determined compounds to the surface of the sensor material,
- the reaction products (7)–(10) from the surface of the sensor material.

The impact of the filter may be due to the crystalline remodelling of the sepiolite and the separation of water during temperature changes. Given that the diameter of a single sepiolite tube ranges from several to several tens of nanometers at ambient temperature, and the cross-section of the oxygen molecules is 26.4 Å^2^, it therefore seems unlikely that the sepiolite layer significantly decreased the rate of diffusion of oxygen to the surface of tin dioxide. Above 550 °C there is a thermal dissociation of tin dioxide according to reaction (11), as stated by the Kröger-Vink notation [23].

(11) $$2SnO_{2}↔2Sn_{Sn}^{x}+2O_{O}^{x}+2V_{O}^{••}+4e^{\prime }+O_{2}↑$$

This reaction gives off oxygen. If the sepiolite layer limited the oxygen diffusion from the layer of tin dioxide into the ambient atmosphere, then the increase of the oxygen concentration would occur in the gas-sensitive layer, which would cause a decrease in the conductivity of SnO_2_; however, the opposite occurs. The analysis of the electrical test results shows that the presence of the sepiolite layer does not also decrease the sensitivity in the presence of VOCs (Figure 11). Below 300 °C, the layer of the sepiolite does not limit the rate of VOC diffusion to the surface, as the sensitivity within this temperature range increases, and above 500 °C it is no longer dependent on the composition of the gas atmosphere. On this basis, it can be assumed that above 300 °C the reconstruction of the microstructure of sepiolite affected the rate of diffusion of the substrates (VOCs) to the surface of the gas-sensitive material. It seems, however, that in the full temperature range, the impact of the sepiolite layer on the rate of diffusion of the determined VOC molecules should be determined. Because of the ionic character of the bonds present in the sepiolite, this layer will have the least impact on the rate of diffusion of the molecules that have the lowest dipole moment, which is demonstrated by the n-butanol molecules (Table 1).

**Figure 13:**
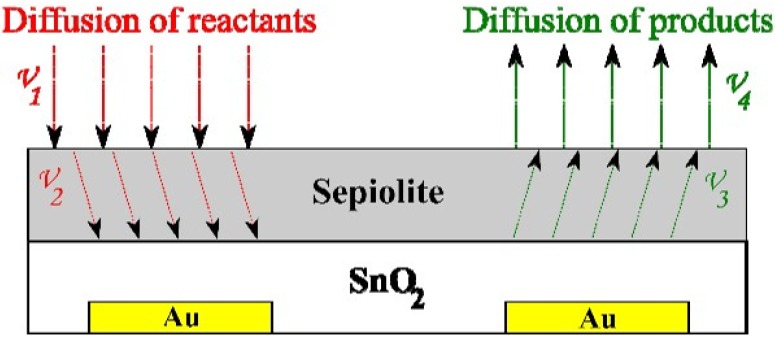
Diffusion of reactants and products to/from the SnO_2_ sensor with the sepiolite filter.

Regardless of the presence or lack of a sepiolite filter, the rate of reactants and product diffusion from atmosphere to the surface of sensor and vice versa is the same (V_1_, V_4_). The filter affects the rate of diffusion, adsorption and desorption of reactants and products (V_2_, V_3_) within a sepiolite layer (Figure 13).

The products of chemical reactions (7)–(10) are carbon dioxide and water. Since a molecule of carbon dioxide is not polar, and the dipole moment of a water molecule is D = 1.85, the structural reconstruction of the sepiolite that occurs under influence of the temperature has a significant effect not only on the sensitivity of the sensor filter in the presence of alcohols, but also on the interaction of the sepiolite (a compound with ionic bonds) with molecules of water.

### 3.4. The Impact of Humidity on the Sensor

**Figure 14:**
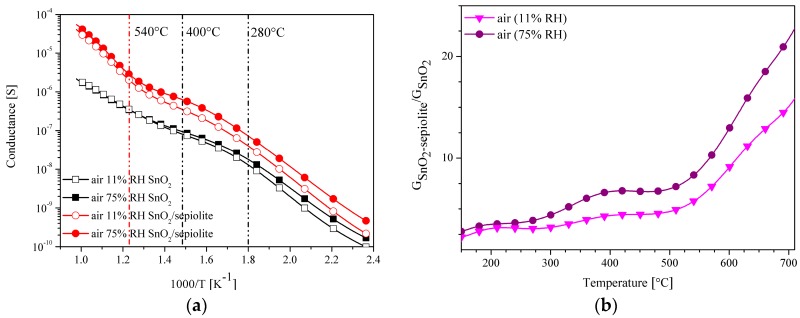
(**a**) Changes in the conductance of SnO_2_ sensor with and without a sepiolite filter as a function of temperature depending on the humidity; (**b**) the relationship of the conductance of the SnO_2_ sensor with the sepiolite filter to the conductance of the sensor without a filter in air, with a relative humidity of 11% and 75%.

The authors carried out the studies using the TSC method of the SnO_2_ sensors with and without a filter in synthetic air with variable humidity. The studies have shown that the conductance of the sensor with the sepiolite filter is approximately two times greater than the conductance of the sensor without a filter below the temperature of 300 °C. (Figure 14a,b).

Above this temperature, this increase becomes more pronounced, but it depends on the moisture, which is particularly evident in Figure 14b, showing the dependence of the sensor conductance with a filter on the conductance of the sensor without a filter. Thus, not only temperature but also humidity have a large impact on the characteristics of the sensor with the sepiolite filter. Water interacts with both the active layer and the sepiolite layer. According to Figure 14a, it can be seen that above 500 °C, water has no impact on the sensor with only the SnO_2_ layer, in contrast to the sensor with an additional layer of sepiolite filter. Considering the effect of water, the influence of oxygen on the detection should also be included (Table 2).

**Figure 15:**
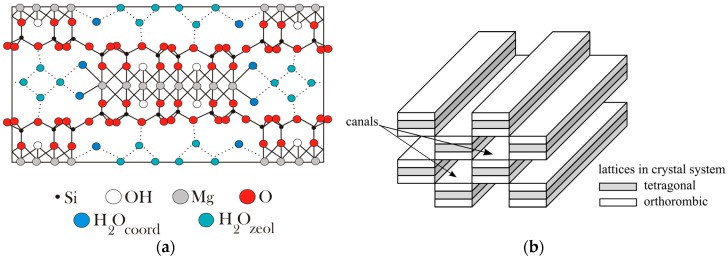
Diagram of the chemical structure of the sepiolite with the adsorbed forms of water (**a**) prepared on the basis of [28]; and (**b**) the channels formed by the combination of unit cells.

The sepiolite, due to the highly developed specific surface and the large number of channels, binds in its structure various forms of water presented in Figure 15 [7,25,27]:

- hygroscopic, formed as a result of the adsorption of H_2_O molecules on the sepiolite surface, with the amount of water depending on the humidity of the air,
- zeolitic, located in the channels of the sepiolite;
- the molecules bound on the boundaries of the layers that have the octahedral system of network nodes;
- structural, bound in the form of hydroxyl groups in octahedral layers.

**Figure 16:**
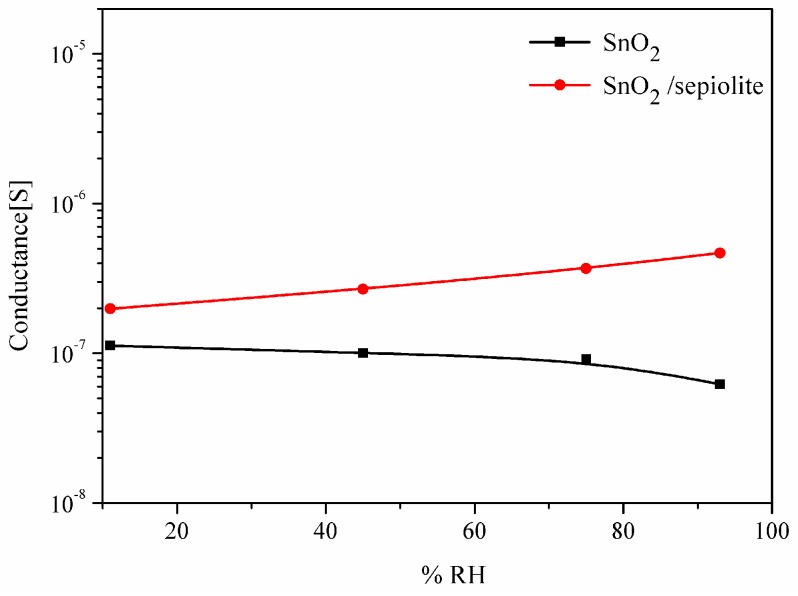
Changes in the conductance of the sensor without a filter and the sensor with a filter as a function of humidity at a temperature of 550 °C.

Various forms of water found in the sepiolite are released at different temperature ranges [7]. First, the molecules of the hygroscopic water are desorbed from the surface of the sepiolite. Then, along with an increasing temperature, zeolitic water is released from the sepiolite (>50 °C) [16]. In the range of 340 °C to 580 °C, water molecules contained in the nodes of the octahedral system are released, and in a temperature range of 780–833 °C, hydroxyl groups are removed from the sepiolite [25]. On the basis of the literature data and the analysis of the test results, the authors believe that the layer of the sepiolite filter not only forms a diffusion barrier for the molecules of the diffusing gas into the sensor material, but also affects the process of the desorption of the reaction products. The water released from the sepiolite at different temperature ranges diffuses not only into the ambient atmosphere, but also into the gas-sensitive material, causing a change in its conductivity. Evidence of this can be seen by the definite change of the optimum operating temperature of the sensor with a filter and an increase in the difference in the conductance sensor with and without a filter at an elevated temperature (Figure 16). Proof of this can be observed in the form of an increase in the sensitivity of the sensors with a filter, particularly in the presence of n-butanol and n-propanol. Most of the water molecules are formed during the oxidation of n-butanol (10), and the least are formed during the determination of methanol (7).

When analysing the impact of the sepiolite layer on the parameters of the resistive gas sensors, the impact of carbon dioxide (CO_2_) produced by the oxidation reaction of hydrocarbons from the measured gas-sensitive layer can be neglected. While some CO_2_ molecules should interact with water molecules adsorbed on the surface of the sepiolite, these effects are specific to temperatures close to the ambient temperature.

## 4. Conclusions

The article presents the impact of the sepiolite layer, which was used as a physical filter, on the electrical parameters of the thick-layered resistive gas sensor. The gas-sensitive layer in the test sensor was made from tin dioxide. The sepiolite was selected as a filter material due to its microstructure. This material consists of tubes with a length of several micrometers, and the diameter of a single tube ranges from several to tens of nanometers. It was expected that the filter layer would limit the diffusion of the aliphatic alcohols with a longer chain. The results of our research showed that the sepiolite layer caused a large increase in the sensitivity of the sensor in the presence of long-chain aliphatic alcohols. On the basis of the electrical tests, it was found that the differences observed in the conductance and the sensitivity of the dual-layer sensors (with a filter) with respect to single-layer sensors (without a filter) are mainly caused by two factors: temperature changes in the structure of the sepiolite and the influence of water, which is separated from the layer of the sepiolite and the chemical processes occurring on the surface of the sensor.

## Figures and Tables

**Table 1:** Chemical formula, dipole moment, relative electric permittivity of the determined volatile organic compounds and sensitivity in the presence of 2 ppm of these compounds.

Compound	Chemical Formula	μ [D]	ε_r_ [-]	Sensitivity	
				without Filter	with Filter
Methanol	CH_3_OH	1.70	33.00	2.45	28.23
Ethanol	CH_3_CH_2_OH	1.69	24.30	3.30	35.04
n-propanol	CH_3_CH_2_CH_2_OH	1.68	20.10	3.48	82.41
n-butanol	CH_3_CH_2_CH_2_CH_2_OH	1.66	17.80	4.48	109.40

**Table 2:** Physical and chemical changes of tin dioxide/sepiolite due to adsorption of oxygen or water on its surface.

Process No.	Material	Temp. [°C]	Physical and Chemical Changes	References
(1)	Sepiolite	>50 °C	desorption of $H_{2}O_{\mathrm{ads}}$ from surface of sepiolite	[16]
(2)	SnO_2_	<130 °C	desorption of $H_{2}O_{\mathrm{ads}}$ from surface of SnO_2_	[24]
(3)	SnO_2_	150 °C	desorption of $O_{2}^{-}$ and transformation of $O_{2}^{-}\to O^{-}$ begins	[20]
(4)	Sepiolite	246 °C	release of zeolitic water	[16]
(5)	SnO_2_	280 °C	decrease the amount of water	[24]
(6)	SnO_2_	400 °C	desorption of water formed from $OH^{-}$	[20]
(7)	Sepiolite	450 and 494 °C	release of water molecules located in nodes of the octahedral system	[25]
(8)	SnO_2_	520 °C	desorption of $O^{-}$	[20]
(9)	SnO_2_	>550 °C	thermal dissociation of SnO_2_	[26]
(10)	Sepiolite	780–833 °C	removal of $OH^{-}$ of sepiolite	[25]
